# Digitalized human resources for health information systems in low- and middle-income countries: a scoping review

**DOI:** 10.1186/s12960-025-01043-x

**Published:** 2025-12-19

**Authors:** Mari Nagai, Raymond Mufwaya-Nsene, Moe Moe Thandar, Sadatoshi Matsuoka, Sumiyo Okawa, Noriko Fujita

**Affiliations:** 1 Bureau of Global Health Cooperation, Japan Institute for Health Security, Tokyo, Japan; 2 Interfaculty Initiative in Planetary Health, Nagasaki University, Nagasaki, Japan; 3 School of Tropical Medicine and Global Health, Nagasaki University, Nagasaki, Japan

**Keywords:** Health workforce, Management information systems, Digital technology, Low and middle income countries, Scoping review

## Abstract

**Background:**

Tracking country-wide human resources for health (HRH) information is a milestone in the global strategy for HRH 2030, and digitalized HRH information systems have been recommended by the World Health Organization. However, the implementation status differs among countries, and most systematic reviews on this topic have been conducted in high-income countries. This scoping review aimed to identify (1) stages of implementation, (2) functional components, (3) facilitators and barriers, and (4) policy impacts or outcomes of digitalized HRH information systems in low- and middle-income countries (LMICs).

**Methods:**

The methodological framework of the Johanna Briggs Institute was used in this scoping review. English articles in two databases (PubMed and Web of Science) with publication dates ranging from inception to August 2023 were gathered, followed by a gray literature search and a reference search. Two author pairs independently performed the study selection. Data were extracted, analyzed, and presented in tabular form alongside a narrative summary.

**Results:**

Forty studies and gray literature from 26 LMICs in Asia and Africa were included in the scoping review. Thirty-three studies and gray literature covered different stages of digitalized HRH information systems’ implementation, including development, pilot, rollout, and maintenance. The HRH registry was the most common, whereas finances and migration were the least common functional components. Thirty-two studies and gray literature reported barriers and facilitators, stratified into four factors and stages. Many barriers were identified in organizational and environmental factors, especially in governance. Interoperability among multiple HRH information systems within a country is the key facilitator, where development partners play a critical role. Sixteen studies and gray literature from nine countries reported positive policy impacts/outcomes. Political commitment, strong national and subnational leadership, and coordination mechanisms among national stakeholders and development partners were key to gaining policy impact.

**Conclusions:**

Barriers and facilitators were common across the studies, and governance factors were particularly crucial at all stages of digitalization. Our stratified methodology for analyzing facilitators and barriers can serve as an analytical framework for evaluating HRH information systems in any country. Data on the private sector and migration could be further strengthened as system components.

**Supplementary Information:**

The online version contains supplementary material available at 10.1186/s12960-025-01043-x.

## Introduction

Human Resources for Health (HRH), one of the six building blocks of the health system defined by the WHO, is an essential component for strengthening health systems and forms the core of healthcare service delivery. Sufficient and equitable distribution of quality HRH responding to societal shifts in health needs—such as pandemic preparedness and response, primary health care, noncommunicable diseases, and aging—is key to achieving Sustainable Development Goals (SDGs) through universal health coverage (UHC) [1–3]. However, there are critical gaps in HRH. According to the World Health Organization (WHO) global strategy on human resources for health, Workforce 2030, the shortage of HRH is over 14 million to achieve the SDG index threshold of 4.45 physicians, nurses, and midwives per 1000 population [4]. Gaps also exist in their distribution, quality, and skill mix worldwide, especially in low- and middle-income countries [5]. A comprehensive assessment of the HRH system is crucial for tackling these gaps [6], and the availability of up-to-date HRH information is fundamental for gap identification, assessment, and usage as evidence for policy decisions [1, 5].

In 2016, the UN General Assembly and World Health Assembly resolved that HRH and HRH information systems were critical for achieving UHC [7]. The HRH information system routinely collects information throughout the life cycle of a multi-cadre HRH [8]. It covers a wide range of information about HRH, including pre-service education, registration, licensure, workplace, skillsets, salaries, continuous professional development, and national and international mobility. Reliable and up-to-date HRH information can provide a comprehensive picture of the current state of HRH in a country, helping policymakers develop, implement, monitor, and assess the impact of political measures against HRH challenges, such as shortages, maldistribution, and low quality. It also contributes to the projection of future workforce requirements that reflect the changing needs of the population.

The Global Strategy for HRH 2030 stipulates that the ability of countries to track the stock, distribution, flow, demand, supply, capacity, and remuneration of HRH is a global milestone [4]. The WHO recommends that member states develop digitalized information systems with minimum datasets to count and document all HRH within national and subnational contexts [8]. However, the implementation stages and data coverage differ from country to country [5]. A systematic review reported factors affecting the implementation process of HRH information systems, with the majority of included studies being conducted in high-income countries [9]. Another systematic review, which included low- and middle-income countries (LMICs) but was not limited to digitalized systems, revealed that while such systems were in place in many LMICs, their functionality and data use for decision-making were often limited [10]. Similar results were identified in case studies that recommended more rigorous future research [11]. Thus, synthesizing common facilitators and barriers to digitalized HRH information systems experienced in LMICs may contribute to the stable and effective utilization of the systems and the realization of the WHO recommendations.

The objective of this scoping review is to identify the following aspects of LMICs: (1) the implementation stages of the digitalized HRH information system; (2) its functional components; (3) facilitators and barriers to its implementation; and (4) policy impacts or outcomes of implementation of the system (i.e., the application of data from the HRH information system into policies).

## Methods

We conducted a scoping review to map the literature on digitalized HRH information systems in LMICs and summarize the available evidence [12]. We applied the methodological framework outlined by the Johanna Briggs Institute (JBI) [13]. The JBI framework has been influenced by the earlier work developed by Arksey and O’ Malley [14]. This review adhered to the Preferred Reporting Items for Systematic Reviews and Meta-Analyses extension for Scoping Reviews (PRISMA-ScR) checklist [15], as indicated in the Supplementary Information (Table S1).

### Identifying the research question

The research questions of this review were as follows:

1. What are the stages involved in the implementation process of digitalized HRH information systems in low- and middle-income countries?
2. What are the functional components of the digitalized HRH information systems, and how are they linked with other health information systems?
3. What barriers and facilitators influence the implementation of digitalized HRH information systems?
4. What are the policy-level outcomes or impacts resulting from the implementation of digitalized HRH information systems?

### Inclusion criteria

Inclusion criteria were identified according to the guidelines outlined by JBI as follows [13].

#### Population

We considered any individuals and entities involved in the digitalization process of the HRH information system as the target population, including government agencies, policymakers, development partners, IT and technology companies, professional associations, non-profit organizations, and healthcare workers.

#### Context

This review focused on studies conducted in low- and middle-income countries.

#### Concepts

The concept of this review was the digitalization of HRH information systems in LMICs, addressing at least one of the four research questions outlined above. Initially, we focused on implementing a digitalized HRH information system at the national level. During the screening and full-text reading of studies, however, we found studies that targeted the subnational level due to the decentralization of HRH administration or the pilot stage for implementing information systems. Although these studies did not target the national level, they contained data relevant to the objectives of our study at the subnational level. Therefore, we included these in the review. We excluded studies on information systems related to disease programs, individual patients, or healthcare services at the health facility or community levels.

#### Types of evidence resources

Qualitative, quantitative, and mixed-method studies published in English were eligible for inclusion. The gray literature was included to capture relevant unpublished studies. Protocols and conference abstracts were excluded because they lacked sufficient detail to permit data extraction. Reviews were also excluded.

### Searching

Two authors (MMT and SO) developed a comprehensive search strategy based on two previous studies [9, 11]. The search strategy was constructed using four key terms reflecting the concepts and context stated above: (1) health, (2) human resources, (3) information systems, and (4) low- and middle-income countries. A preliminary search of PubMed was conducted. The text words in the titles and abstracts of relevant articles were used to develop a comprehensive search strategy, which was refined through the authors’ discussion. A second search was undertaken on the Web of Science after modifying the search terms to match those in the database. We did not impose any date restriction on the search. We searched for potential papers published before July 7, 2023, and August 22, 2023, in PubMed and Web of Science, respectively. Following JBI’s scoping review guideline, we performed a search of potential gray literature published before 31 August 2023, using both Google and Google Scholar, following the simplified search strategy we used for PubMed. We also performed a reference search of cited publications within potential studies and previous systematic reviews.

### Study selection

Following the search, identified articles were first imported into the reference management software EndNote. After removing duplicates, the remaining articles were imported into the online reference management software, Covidence (Veritas Health Innovation, Melbourne, Australia). Two author pairs (MN, MMT, NF, RM, SM, and SO) independently screened the titles and abstracts using the pre-defined inclusion and exclusion criteria. Studies with conflicting screening results were resolved through discussions among the six authors. The full texts were retrieved and uploaded to Covidence. Similarly, two author pairs independently read the full text of the potentially eligible studies, evaluated their eligibility, and resolved disagreements through discussion.

### Extracting and charting the data

We created a standardized data extraction form using Microsoft Excel, and the following information was extracted: country, region, the first author’s name, year of publication, study design, digitalized HRH information systems (name of database, name of software, ownership, development partner, starting year, stage covered in the paper, and coverage of private or public health facilities), functional components in the existing HRH information systems, and linkage within HRH information systems and with other health information systems, barriers to and facilitators for the implementation of HRH information systems, and the impacts or outcomes of the implementation of the system. Data extraction was performed independently by six authors (MN, MMT, NF, RM, SM, and SO), and the extracted data were verified by another author. The disagreements in data extraction were resolved through discussions among the six authors.

### Data synthesis and presentation of the results

The 11 components suggested by WHO were used to categorize the functional components in the existing HRH information systems per country: HRH registry (i.e., central repository of data on the HRH), training (i.e., in-service professional development), payroll (i.e., salaries, bonuses, and other financial incentives for HRH), retirement (i.e., retirement age, pension plans, and benefits upon retirement), workforce production (i.e., pre-service education, and management of educational institutions), vacancy and recruitment (i.e., status of vacant positions, the recruitment process), registration and licensing (i.e., registration and licensing of HRH), benefits (i.e., health insurance, housing, and other incentives), performance management (i.e., performance assessments of HRH, feedback mechanisms, and performance improvement plans), migration (i.e., out-migration and in-migration of HRH), and finance (i.e., budget allocation, expenditure at national, sub-national, facility level, not individual level) [8]. In addition to the 11 components, we reviewed the linkages within multiple HRH information systems and with other health information systems irrelevant to HRH, such as DHIS 2.

We stratified barriers and facilitators for the implementation of HRH information systems according to four factors: information technology/infrastructure (i.e., hardware, software, office space, electricity, internet), individual (i.e., skills, willingness, attitudes), organizational (inner factors such as governance and technical aspects within the HRH information systems stakeholders), and environmental (external factors such as political, economic, and social contexts) [9, 11, 16, 17]. We then classified the barriers and facilitators in each factor into four implementation stages based on our interpretation of the study’s information: development (i.e., conception, architecture design, provision of hardware, and development of software), pilot (i.e., operationalization of the system in a limited target or geographic area before wider scaling up), rollout (i.e., scaling up to a wider target or nationwide), and maintenance (i.e., sustainable utilization of the system) [18]. Those not specific to any stage were categorized as having non-specific stages. Finally, we summarize the major policy impacts/outcomes of the implementation of the system (i.e., reflection of analysis results of the data from the HRH system in HRH policies).

## Results

### Search results

**Fig. 1:**
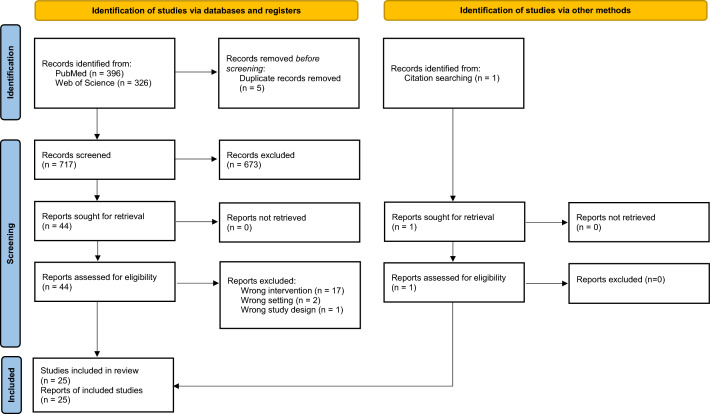
PRISMA flow diagram for study selection

A total of 722 records were identified through the database searches, and 112,014 records through the gray literature search. After removing 111,818 duplicates (5 from the database search and 111,813 from the gray literature search that were automatically removed by the Google algorithm), 918 records (717 from the database search and 201 from the gray literature search) were eligible for screening titles and abstracts. Of these, 854 were excluded after careful examination following the inclusion criteria. The majority of these studies and gray literature focused on information systems related to disease programs, individual patients, or healthcare services at the health facility or community levels. Following a full-text review, 26 studies and gray literature were excluded, leaving 38 studies and gray literature. Additionally, one study and one gray literature were identified through a reference search and were included. Finally, 40 studies and gray literature were included in this review. Figure 1 illustrates the study selection process following the PRISMA guidelines.

### Characteristics of included studies

The included studies and gray literature, published between 2007 and 2023, were sorted by country (Table 1). Most studies were published within the last decade. Eleven studies adopted qualitative methods, ten used quantitative methods, and seven used mixed methods. Moreover, eight reports, two policy/technical briefs, one thesis, and one presentation slide were also included. All the studies and gray literature were conducted in LMICs across Africa and Asia. Nine reported data on Kenya [19–27], seven on Uganda [28–34], four on Iran [35–38], three on Tanzania [18, 39, 40], three on Mozambique [34, 41, 42], two on Bangladesh [43, 44], two on Nigeria [45, 46] and one each from Burkina Faso [34], the Democratic Republic of Congo (DRC) [47], Ethiopia [48], Indonesia [49], Malawi [50], Pakistan [51], South Africa [52], Sudan [53], Swaziland [54], Zambia [55], Zimbabwe [56], and WHO South-East Asia region countries (SEARC) [57].

**Table 1:** Characteristics of the included studies

Country (region)	Author and publication year (reference number)	Study design	Digitalized human resources for health information system					
			Name of the system/database and software	Ownership	Development partner	Starting year	Stage covered in the paper	Coverage level and sectors
WHO South-East Asia region countries (SEARC): Bangladesh, Bhutan, India, Indonesia, Maldives, Myanmar, Nepal, Sri Lanka, Thailand. Timor Leste (Asia)	Cometto 2019 [57]	Quantitative (Cross-sectional)	Human resources for health information system in 10 countries (not all databases were digitalized) Not specified software	Ministry of Health (MoH)	Funding from domestic, overseas development or mixed sources	Not specified	Not specified	National level Public and private
Bangladesh (Asia)	Alam 2016 [43]	Quantitative (Cross-sectional)	Not specified database Not specified software	Not specified	Not specified	Not specified	Not specified	Level unclear Private
Bangladesh (Asia)	Bhattacharyya 2020 [44]	Qualitative (Desk review, key informant interviews, and in-depth interviews)	Human resource information system, including Geo-location Registry, Facility Registry, Posts Registry, Provider Registry Not specified software	Ministry of Health and Family Welfare (Directorate General of Health Services)	Not specified	2018	Rollout	National level Public
Burkina Faso (Africa)	Vital Wave, IntraHealth, and Cooper/Smith (2021) [34]	Report	Council registries; SIGASPE; MoCs; DIAN; ALIAS; Health workforce monitoring framework; LogRH; eGratuite des Soins; ENDOS (DHIS2)	Professional councils; Ministry of Economy Finance; Ministry of Civil Service; MoH-HR Unit; and MoH	Not specified	Not specified	Not specified	National level Public and private
The DRC (Africa)	Likofata Esanga 2017 [47]	Mixed methods (Data collection and interviews)	Human resource information system Software: iHRIS Manage	Ministry of Public Health; Ministry of Public Service; Ministry of Finance	IntraHealth	2015	Pilot	Subnational level Public
Ethiopia (Africa)	Dilu 2017 [48]	Mixed methods (Questionnaire, in-depth interview, and observation checklists)	Human resource information system Not specified software	Federal Ministry of Health (Human Resource Development Directorate; Food, Medicine and Health Care Administration and Control Authority)	Not specified	Not specified	Pilot	Subnational level Public
Indonesia (Asia)	HRH2030 2019 [49]	Report	Multiple databases by multiple ownerships Not all databases are specified MoH owns 4 databases: SI-SDMK, PUSRENGUN, PUSDIK SDMK, PUSLAT SDMK, Not specified software	Ministry of Research and Higher Education; MoH; Ministry of Manpower; Ministry of Home Affairs	USAID and PAPFAR	Not specified	Maintenance	National level Public and private
Iran (Asia)	Ehsani-Chimeh 2018 [35]	Quantitative (Cross-sectional)	AZERAKHSH Not specified software	Ministry of Health and Medical Education	Not specified	Not specified	Rollout	National level Public
Iran (Asia)	Najafpour 2022 [36]	Qualitative study (Interviews)	Multiple databases Not specified software	Multiple databases maintained by various organizations	Not specified	Not specified	Development	Level unclear Public and private
Iran (Asia)	Zhila 2022 [37]	Mixed methods (Assessment with checklists and key informant interviews)	Multiple databases (not all databases were digitalized) Not specified software	Ministry of Health and Medical Education; Medical science universities; Hospitals	Not specified	Not specified	Development	Level unclear Public and private
Iran (Asia)	Najafpour 2023 [38]	Mixed methods (Literature review, qualitative phase, and expert panels)	Not specified database Not specified software	Not specified	Not specified	Not specified	Development	Level unclear Public and private
Kenya (Africa)	Riley 2007 [19]	Quantitative study (Secondary data analysis)	Kenyan nursing database Not specified software	Nursing Council of Kenya (NCK); MoH; Privately employed worksite	Emory University; US Centers for Disease Control and Prevention (CDC); President’s Emergency Plan for AIDS Relief (PEPFAR)	2002	Development and rollout	National level Public and private
Kenya (Africa)	Waters 2013 [20]	Mixed methods (Interviews and secondary data analysis)	Kenya Health Workforce Information System (KHWIS) databases Not specified software	Government of Kenya; NCK: Kenya Medical Practitioners and Dentists Board; Kenya’s Clinical Officers Council; Kenya Medical Laboratory Technicians and Technologists Board	Emory University; CDC; PEPFAR	2002	Rollout	National level Public and private
Kenya (Africa)	Wakaba 2014 [21]	Quantitative study (Secondary data analysis)	Regulatory Human Resource Information System (rHRIS); KHWIS Not specified software	NCK; MoH	Emory University; CDC; PEPFAR	2002	Rollout	National level Public and private
Kenya (Africa)	Appiagyei 2014 [22]	Mixed methods (Secondary data analysis and key informant interviews)	rHRIS; KHWIS Not specified software	NCK; MoH	Not specified	Not specified	Rollout	National level Public and private
Kenya (Africa)	Oluoch 2015 [23]	Quantitative (Observational study)	rHRIS; Integrated Human Resources Information System (iHRIS); District Health Information Software-2 (DHIS2); Master Facility List (MFL, a registry of health facilities) Software: iHRIS; DHIS2; MFL	NCK; MoH	Not specified	2002	Not specified	National level Public and private
Kenya (Africa)	Kenya Health Workforce Project (2015) [25]	Report	Not specified database Software: iHRIS	NCK; MoH	Emory University; CDC; PEPFAR	Not specified	Development, Roll out, and Maintenance	National level Public and private
Kenya (Africa)	Yumbya (2016) [26]	Presentation slides	Not specified database Software: iHRIS	Not specified database Software: iHRIS	Emory University; CDC; PEPFAR	Not specified	Development, Roll out and Maintenance	National level Public and private
Kenya (Africa)	Thuku 2020* [24]	Qualitative (Case study)	Not specified database Software: iHRIS	Not specified database Software: iHRIS	Not specified	Not specified	Not specified	Level unclear Not specified
Kenya (Africa)	Thuku 2021 [27]	Policy brief	Not specified database Software: iHRIS	Not specified	Not specified	Not specified	Maintenance	Level unclear Not specified
Malawi (Africa)	Sathamkamwa (2022) [50]	Thesis	Malawi National Health Information System Software: iHRIS Plan; iHRIS Train; iHRIS Qualify; iHRIS Retain; iHRIS Manage	Ministry of Health	USAID; CDC; PEPFAR	2005	Development	National level Public and private
Mozambique (Africa)	Waters 2016 [41]	Qualitative (Case study)	Mozambican human resource information system named "eSIP-Saude" (electronic Personnel Information System for Health) Software: developed by the Center for Development of Financial Information Systems technical staff	MoH; Ministry of State Administration and Public Function; Ministry of Economy and Finance	CDC; PEPFAR; Jhpiego; Belgian Technical Cooperation	2010	Development and rollout	National level Public
Mozambique (Africa)	Vital Wave, IntraHealth, and Cooper/Smith (2021) [34]	Report	Membership registries; National payroll system (eFOLHA); Financial management information system (eSISTAFE); National public employee MIS (eCAF); New system with longitudinal record (eSNGRH); eCAF-extension; Spreadsheet 7; eSNGRH-extension, Pre-service training (SIFo); In-service training (SIFIn), Master facility list; Health Management Information System (SISMA) Not specified software	Professional councils; Ministry of Finance; Ministry of Public Administration and Civil Service; MoH-HR Unit; MoH	Not specified	Not specified	Rollout	National level Public and private
Mozambique (Africa)	Fernandes 2023 [42]	Quantitative study (Secondary data analysis)	eSIP-Saude	MoH	Not specified	Not specified	Rollout and maintenance	National level Private and public
Nigeria (Africa)	Okoroafor 2022 [45]	Qualitative (Case study)	Nigeria Health Workforce Registry (NHWR) Software: iHRIS Manage	Federal Ministry of Health	World Health Organization; Global Health Canada	2015	Development, rollout, and maintenance	National level Public and private
Nigeria (Africa)	USAID Health Workforce Management 2023 [46]	Technical brief	NWWR Software: Kobo Toolbox	Not specified	Not specified	2022	Development and maintenance	Subnational level Not specified
Pakistan (Asia)	Kumar 2013 [51]	Quantitative (Cross-sectional)	Multiple databases (not all databases were digitalized) Not specified software	Public sector (district/provincial health offices; tertiary level care hospitals; DHIS cell; primary health care centers); Private institutions (hospitals; international Non-governmental Organizations (NGOs); donors)	Not specified	Not specified	Not specified	Subnational level Public and private
South Africa (Africa)	MoH, South Africa (2020) [52]	Report	Multiple databases including Personal salary administration system and several health professional registries Not specified software	National Tracery; Health professional councils	Not specified	Not specified	Development	Level unclear Private and public
Sudan (Africa)	Badr 2013 [53]	Qualitative (Case study)	Human Resources for Health (HRH) information system; HRH observatory Not specified software	MoH; Ministry of Higher Education; Governmental institutions; Health worker registration councils; Professional associations; NGOs	Global Health Workforce Alliance	2006	Development (implement HRH policy)	National level Public and private
Swaziland (Africa)	Ministry of Health, Swaziland (2012) [54]	Report	Human Resource Information System (HRIS)	MoH	Global Fund; USAID; PEPFAR; World Bank; WHO; UNICEF	2006	Development, rollout, and maintenance	National level Public and private
Tanzania (Africa)	Ministry of Health and Social Welfare (2014) [39]	Report	Human Resource for Health Information System (HRHIS); Training Institution Information System (TIIS) Not specified software	Ministry of Health and Social Welfare	Japan International Cooperation Agency (JICA)	Not specified	Development, pilot, rollout, and maintenance	National level Public and private
Tanzania (Africa)	Ishijima 2015 [18]	Qualitative (Case study)	HRHIS; TIIS Software: Web-based for HRHIS; Web and enterprise-based for TIIS	Ministry of Health and Social Welfare	JICA	2009	Development, pilot, rollout, and maintenance	National level Public and private
Tanzania (Africa)	Ministry of Health, Community Development, Gender, Elderly and Children (2021) [40]	Report	HRHIS; TIIS Not specified software	Not specified	Not specified	Not specified	Maintenance	National level Not specified
Uganda (Africa)	Spero 2011 [28]	Quantitative (Secondary data analysis)	HRHIS (this paper focused on the database of Uganda Nurses and Midwives Council (UNMC)) Software: iHRIS Qualify	MoH; UNMC; Uganda Medical and Dental Practitioners Council; Allied Health Professional Council (AHPC); Uganda Pharmacy Council	United States Agency for International Development (USAID); IntraHealth	2005	Rollout	National level Public and private
Uganda (Africa)	Ministry of Health (MoH) (2014) [31]	Report	HRHIS (115 databases: Pre-service train including Ministry of Education and Sports (MoES), Uganda Nurses and Midwifery Examination Board (UNMEB), and Health Tutors College (HTC); In-service train including MoH Human Resources Development (HRD) and MoH Human Resources Management (HRM); Qualify including UNMC, Uganda Medical and Dental Professionals Council (UMDPC), Pharmacy Council and Uganda Pharmaceutical Society, and AHPC; Manage including MoH–HRM, Two National Referral Hospitals, 14 Regional Referral Hospitals, 14 Regional Referral Hospitals, Uganda Blood Transmission Services, National Medical Stores, Uganda Cancer Institute, 32 Uganda Catholic Medical Bureau, 25 Uganda Protest Medical Bureau, and 92 districts) Software: iHRIS Manage: iHRIS Qualify; iHRIS Train	Ministries, councils, professional societies, hospitals, bureaus and districts mentioned in the database column	USAID; IntraHealth	2005	Development and rollout	National level Public and private
Uganda (Africa)	Driessen 2015 [29]	Qualitative (Case study)	HRHIS (this paper focused on databases of AHPC, Health Tutors College (HTC), MoH Human Resources Development (HRD), MoH Human Resources Management (HRM), Uganda Nurses and Midwives Examination Board (UNMEB), Uganda Protestant Medical Bureau (UPMB)) Software: iHRIS Manage: iHRIS Qualify; iHRIS Train	MoH; AHPC; UNMEB; UPMB	USAID; IntraHealth	2005	Rollout	National level Public and private
Uganda (Africa)	Larsen 2015 [32]	Qualitative (Case study)	HRHIS (UMDPC, UNMC, Pharmacy Council, AHPC); 2 National hospitals; 13 regional referral hospitals; 69	MoH; four health professional councils; hospitals; and districts	USAID; IntraHealth	Not specified	Rollout	National level Public and private
Uganda (Africa)	Jamiru 2019 [33]	Qualitative (Key informant interviews)	Not specified database Software: iHRIS Manage: iHRIS Qualify; iHRIS Train	Not specified	Not specified	Not specified	Rollout	National level Public
Uganda (Africa)	Vital Wave, IntraHealth, and Cooper/Smith (2021) [34]	Report	Not specified database Software: iHRIS Manage: iHRIS Qualify; iHRIS Train	Not specified	USAID, the European Union, WHO, UNICEF, IntraHealth	2006	Rollout and maintenance	National level Public and private
Uganda (Africa)	Mansour 2022* [30]	Mixed methods (Questionnaires and focus group discussion)	Not specified database and software	Not specified	Not specified	Not specified	Not specified	Level unclear Not specified
Zambia (Africa)	Were 2019 [55]	Qualitative (Case study)	Health workforce information system Software: developed by Kenyan and Zambian technical teams	Health Professionals Council of Zambia; General Nursing Council of Zambia; Ministry of Community Development, Mother and Child Health; Ministry of Health; Zambia’s health training institutions	Emory University; CDC; PEPFAR; Kenyan consulting team	2015	Development and rollout	National level Private
Zimbabwe (Africa)	Waters 2017 [56]	Quantitative (Gap analysis)	Zimbabwe Human Resource Information System Software: MySQL	Ministry of Health and Child Care; Regulatory councils (Medical and Dental Council of Zimbabwe; Nursing Council of Zimbabwe)	Emory University; CDC; PEPFAR	2009	Not specified	National level Public and private

*Thuku 2020 and Mansour 2022 did not report information on the HRH information system. However, other information was valuable for identifying barriers and facilitators of the digitalization of the HRH information system. Thuku (2020) reported on how Kenya established a national HRH Inter-agency Coordinating Committee. Mansour 2022 reported the decision space available to District Health Management Teams for human resource management functions in Uganda

### Digitalization of HRH information system

In general, the purpose of digitalizing HRH information systems was to create a computerized database system for HRH, which was expected to provide accurate and up-to-date data for the appropriate allocation of HRH resources and to inform health policy and evidence-based decision-making. Such digitalized HRH information systems were implemented at the national level (reported in 28 studies and gray literature) and subnational level (province or state) (reported in 4 studies and gray literature). Eight studies and gray literature did not clearly indicate the level of implementation. These systems were owned by the government sector, such as the Ministry of Health, the non-government sector (health professional council/private hospitals/NGOs), or both. Typically, government-run systems exclusively cover health workers in the public sector, whereas systems under non-governmental ownership also cover health workers in the private sector. Notably, in Nigeria and Tanzania, government-owned systems have extended their coverage to health workers in the private sector. Kenya introduced the system in 2002, which was the earliest among those reported, followed by Malawi and Uganda in 2005, Swaziland and Sudan in 2006, and Zimbabwe in 2009. Mainly, African countries had development partners for the digitalization of the system, including Belgian Technological Cooperation, Emory University, European Union, Global Fund, Global Health Canada, Global Health Workforce Alliance, IntraHealth, Japan International Cooperation Agency (JICA), Jhpiego, United Nations Children’s Fund (UNICEF), United States Agency for International Development (USAID), United States Centers for Disease Control and Prevention (CDC), United States President’s Emergency Plan for AIDS Relief (PEPFAR), World Bank, and WHO. Among African countries, the DRC, Kenya, Malawi, Nigeria, and Uganda utilized iHRIS software, an open-source software developed by IntraHealth; meanwhile, Tanzania and Zimbabwe developed the software in-house. There was no information available about development partners, either technical or financial, in Asian studies, except in the case of Indonesia.

### Stages of the implementation process

Thirty-three studies and gray literature covered different stages of digitalized HRH information systems implementation, including development, pilot, rollout, and maintenance. The remaining studies and gray literature did not provide specific information regarding these implementation stages. Studies from the DRC, Ethiopia, Malawi, South Africa, and Sudan reported that the systems were either in the development or pilot stages. In Kenya, Mozambique, Nigeria, Swaziland, Tanzania, Uganda, and Zambia, digitalized systems have been rolled out either nationwide or in specific regions. In Indonesia, all four HRH information systems owned by the Ministry of Health are in the maintenance stage. One study from Iran indicated that the HRH information system, intended to cover the public sector and owned by the Ministry of Health, was in the rollout stage. In contrast, three studies from Iran reported the existence of over 30 HRH databases across both the private and public sectors, all of which were in the development stage. One gray literature from Uganda reported that 115 databases were included in HRHIS [31]. In Bangladesh, one study reported the nationwide rollout of a system owned by the Ministry of Health to cover the public sector, whereas another emphasized the need to extend coverage to the private sector.

### Functional components of digitalized HRH information system and linkage with other health information systems

Table 2 shows an overview of the functional components of HRH information systems and their linkages with other health information systems in the country, which we could confirm from the eligible papers. We did not include the study from SEARC that included 10 countries [6] because it did not provide information on country-specific functional components. As a result, 18 countries were included: Bangladesh, Burkina Faso, the DRC, Ethiopia, Indonesia, Iran, Kenya, Malawi, Mozambique, Nigeria, Pakistan, South Africa, Sudan, Swaziland, Tanzania, Uganda, Zambia, and Zimbabwe. Of the 11 functional components, the HRH registry was the component that most countries utilized in any of their HRH information systems (n = 13), followed by training (n = 11), payroll (n = 11), retirement (n = 8), workforce production (n = 8), vacancy and recruitment (n = 8), registration and licensing (n = 7), performance management (n = 6), and benefits (n = 5). Finance and migration were the components utilized by the fewest number of countries (n = 3). Six countries—Bangladesh, Kenya, Mozambique, Swaziland, Tanzania, and Uganda—mentioned that their HRH information systems were linked with other health information systems (e.g., facility registries, district health information systems). On the other hand, we found that most studies did not provide detailed information on the interoperability of functional components within HRH information systems. Although no clear descriptions exist, some countries may have comprehensive or partial interoperability within HRH information systems.

**Table 2:** Overview of digitalized HRH information system per country: functional components and linkage with other health information systems

Country	HRH registry	Training	Payroll	Retirement	Workforce production	Vacancy and recruitment	Registration and licensing	Performance management	Benefits	Migration	Finance	Linkage with other health information systems*
Bangladesh [43, 44]	✔	✔		✔		✔						✔
Burkina Faso [34]			✔						✔			
DRC [47]	✔		✔									
Ethiopia [48]												
Indonesia [49]	✔	✔	✔	✔	✔	✔	✔	✔	✔			
Iran [35–38]	✔	✔	✔	✔	✔	✔	✔	✔	✔			
Kenya [19–27]	✔	✔	✔	✔	✔		✔	✔		✔		✔
Malawi [50]		✔	✔		✔	✔	✔	✔				
Mozambique [34, 41, 42]	✔	✔	✔	✔	✔	✔						✔
Nigeria [45, 46]	✔											
Pakistan [51]	✔	✔	✔									
South Africa [52]	✔		✔									
Sudan [53]		✔			✔					✔		
Swaziland [54]	✔			✔		✔						✔
Tanzania [18, 39, 40]	✔	✔	✔		✔		✔				✔	✔
Uganda [28–31, 34]	✔	✔	✔	✔	✔	✔	✔	✔	✔	✔	✔	✔
Zambia [55]							✔					
Zimbabwe [56]	✔	✔		✔		✔		✔	✔		✔	
Counting	13	11	11	8	8	8	7	6	5	3	3	6

*Linkage with other health information systems means that the country’s HRH information systems have linkage with the following system: Geo-location Registry, Facility registry (Bangladesh), Ministry of Public Service database (Swaziland), Master Facility List, District Health Information System (Kenya), Health Management Information System (Tanzania), Master Facility List, Health Management Information System (Mozambique), and system not specified in the reference (Uganda)

### Barriers and facilitators of the digitalized HRH information systems’ implementation

Of the 40 studies and gray literature, 32 reported multifaceted barriers and facilitators that influenced the implementation of HRH information systems. We categorized them into four factors (information technology [IT] and infrastructure, individual, organizational, and environmental) and then within four stages (development, pilot, rollout, and maintenance), or as being non-stage-specific, as summarized in Table 3.

**Table 3:** Barriers and facilitators for the implementation of the digitalized HRH information systems

Stage	Barriers/weaknesses/threats	Facilitators/strengths/opportunities
Information Technology (IT)/Infrastructural factors		
Not stage-specific	- Inadequate office space or IT hard component. *Pakistan *[51]*, Uganda *[33]*, SEARC *[57] - Limited IT platform, including software and networking. *Bangladesh *[43] - Unreliable internet or electricity. *Pakistan *[51]*, Uganda *[33]*, SEARC *[57] - Lack of maintenance. *Pakistan *[51]	- Specific office space and adequate IT hardware. *Kenya *[20]*, Pakistan *[51] - Adequate IT platform, including software and networking. *Tanzania *[18]*, Mozambique *[41]*, Uganda *[28] - Reliable internet or electricity. *Kenya *[20] - Local maintenance. *Kenya *[20]*, Tanzania *[18] - Integration or interoperability of the multiple HRH information systems’ software. *Mozambique *[41]*, Tanzania *[18]*, Uganda *[29]
Development stage	–	- Establishing an interoperability approach. *Tanzania *[18]
Pilot stage	- Inadequate office space or IT hard component. *Ethiopia *[48] - Unreliable internet or electricity. *Ethiopia *[48]*, Tanzania *[18]	- Usage of Dropbox to share and store the HR information system’s related technical instructions and training tools. *Uganda *[31]
Rollout stage	- Unreliable internet or electricity. *Tanzania *[18, 39]* Uganda *[31]	–
Maintenance stage	- System failure resulting from the limited data-handling capacity of the Microsoft Access-based HRH information platform. *Swaziland *[54]	–
Individual factors		
Not stage-specific	- Poor adoption of HRH information systems by the human resource/information system manager. *Bangladesh *[43] - Lack of motivation to use IT. *SEARC* [57], *Tanzania *[39] - Low level of IT literacy of system users. *Iran *[38]*, Pakistan *[51]*, SEARC *[57] - High turnover of leadership positions in the HRH units. *SEARC *[57] - Shortage of skilled staff/IT staff to maintain the HRH information system, including IT infrastructure. *Pakistan *[51], *Kenya *[26]	- Positive attitudes toward the HRH information system among users. *Tanzania *[18]—Willingness to learn and commitment of system users. *Tanzania *[18], *Zambia *[55] - Participation in training and capacity building. *Mozambique *[41]*, Uganda *[28]
Development stage	–	- Orienting the system users to the fact that the system is part of their routine work. *Tanzania *[18] - Involvement of the system users in the field. *Tanzania *[18]*, Mozambique *[41]
Pilot stage	- Lack of dedication from the top management. *Ethiopia *[48] - Lack of skilled staff for computer literacy. *Ethiopia *[48]*, Tanzania *[18] - Lack of motivation to use IT. *Ethiopia *[48]	–
Rollout stage	- High turnover of leaders. *Kenya *[24] *-* High turnover of trained staff. *Uganda *[31] - Lack of a critical mass of skilled staff to run the HRH information systems. *Uganda *[30] - Minimal familiarity with HRH information systems among HR managers. *Tanzania *[39]	- System users’ participation in training & capacity building. *Tanzania *[18] *-* Training resource persons to deliver technical support to the district and hospitals. *Uganda *[31]
Maintenance stage	- Lack of understanding/training on the HRH information systems. *Indonesia *[49]	- Participation in training on how to utilize data & maintain IT infrastructures. *Tanzania *[18] - Incentives for complete, up-to-date, and accurate data. *Indonesia *[49] - Skilled staff to handle data entry burden. *Indonesia *[49]
Organizational factors		
Not stage-specific	Governance aspect *Structural weakness:* - Unclear functional responsibilities with a weak regulatory framework. *SEARC *[57]*, Iran *[37, 38] - Lack of ownership and buy-in among some stakeholders. *Kenya *[26] - Lack of active involvement by HR managers, with IT teams leading system use. *Uganda* [33] - No effective policy intervention to increase the staff with IT literacy to manage the HRH information systems. *SEARC *[57]*, Iran *[38]*, Pakistan *[51] - Lack of a comprehensive strategic plan for HRH information systems. *Iran *[38] - Lack of guidelines, protocols, and standardized tools. *SEARC *[57]*,* the DRC [47], *Iran *[38]*, Nigeria *[45]*, Pakistan *[51] - Inadequate data validation mechanism. *SEARC *[57] *-* Multiple, fragmented, stand-alone systems with no unified method of uniquely identifying health workers, limiting interoperability. *Uganda *[33, 34] *-* Multiple systems requiring different login credentials resulted in user fatigue, leading to reduced use of data. *Uganda *[34] - Insufficient human resources in the MoH to update the National Health Workforce Registry. *Nigeria *[46] *Process weaknesses:* - Weak top-down communication, monitoring & evaluation mechanisms. *SEARC *[57]*, Iran *[38]*, Pakistan *[51] - Lack of communication, cooperation, and coordination among concerned parties for integration/interoperability of HRH information systems. *SEARC *[57]*, DRC *[47]*, Nigeria *[45]*, Kenya *[23] - Weak communication and leadership to standardize the broad and fragmented HRH databases. *Iran *[36, 38] - Reluctance/lack of cooperation of the private sector or other stakeholders for data sharing. *Iran *[37, 38]*, Kenya *[21]*, Nigeria *[45]*, Pakistan *[51]*, Sudan *[53] *Capacity weakness:* - Lack of capacity to manage HRH information systems. *Pakistan *[51] - Inadequate capacity of managers in regulatory bodies to use data. *Kenya *[26] Technical aspects: *Weakness* - Poor quality/Incomplete data. *Nigeria *[45]*, Tanzania *[18]*, Zimbabwe *[56], Uganda [33] - Erratic HRH data reporting and dissemination. *Pakistan *[51] - Not updated HRH data/statistics for publication. *SEARC *[57] - No linkage between the Nursing workforce database owned by the Nursing Council with the MOH worksites’ staff data, as well as salary and benefits components. *Kenya *[19]	Governance aspect *Structural Strengths:* - Existence of HRH unit/governance structure at national and sub-national levels. *SEARC *[57]*, Bangladesh *[44]*, DRC *[47]*, Kenya *[19]*, Mozambique *[41]*, Nigeria *[45]*, Sudan *[53]*, Tanzania *[18]*, Uganda *[30] - HRH information system linked and built upon existing MoH/Government owned system and infrastructure. *DRC *[47]*, Nigeria *[45]*, Mozambique *[41]*, Tanzania *[18] - Strong leadership of stakeholders on HRH information system. *Iran *[36, 38]*, Kenya *[24]*, Nigeria *[45]*, Sudan *[53]*, Uganda *[28]*, Zambia *[55] - Policy makers’ involvement in the HRH governance structures. *Nigeria *[45] - Well-developed strategic plan of HRH information systems. *Nigeria *[45] *Process strengths:* - Coordination forum/communication mechanism between national & sub-national levels was developed. *Kenya *[24], *Kenya* [26] - Rules or mechanisms to assure interoperability of the HRH information systems were agreed. *Kenya *[23] - Accountability norms were developed. *Sudan *[53] - Standardized dataset was agreed and guidelines were developed. *Nigeria *[45] - Development and continuous monitoring of a detailed sustainability and transition plan for agency ownership. *Kenya *[26] *Capacity strengths:* - Supportive supervision to lower-levels. *Mozambique *[41]*, Nigeria *[45]*, Uganda *[30] - Close follow-up and supportive supervision by a team composed of MoH staff and system developer. *Tanzania *[18] *-* Mentorship for new agencies. *Kenya *[26] *-* Stimulating data use by national and subnational regulatory agencies through regional best practice forum participation. *Kenya *[26] Technical aspects*:* *Strengths* - Existence of local experts. *Tanzania *[18] - Existence of a quality control center unit/institution. *Iran *[38] - Annual publication of the HRHIS-generated reports to guide decision-makers and policy formulators. *Nigeria *[45] - Public availability/Publication of HRH statistics from HRH information systems. *SEARC *[57]*, Nigeria *[45]
Development stage	–	Governance aspect *Structural strengths:* - Local government leadership & engagement. *Mozambique *[41]*, Tanzania *[18] - Involvement of relevant sections and departments in the Ministry of Health. *Mozambique *[41]*, Tanzania *[18]*, Kenya *[26] *Process strengths:* - Prior situation analysis/review. *Iran *[36, 38]*, Mozambique *[41]*, Nigeria *[45]*, Tanzania *[18]*, Zambia *[55]*. Kenya *[26] Technical aspect *Strength:* - Using a national identification as an HRH identifier. *Iran *[36] *-* Align and streamline the HRH information system’s design with existing boards/councils’ organizational structures and functions*. Kenya *[26]
Pilot stage	Governance aspect *Process weaknesses:* - Lack of teamwork among departments. *Ethiopia *[48] - Lack of cooperation of facilities and institutions in pilot sites in data sharing. *Tanzania *[18] - Poor stakeholders’ commitments. *Ethiopia *[48]	Governance aspect *Process strengths:* - Providing detailed explanations and dissemination of the system. *Tanzania *[18]
Rollout stage	Governance aspect *Structural weakness:* - Limited authority of the district health management team to manage the HRH information system in the local context. *Uganda *[30]	Governance aspect *Process strengths:* - Involvement of provincial stakeholders through inter-county/provincial HRH forum. *Kenya *[24] - Prior situation analysis to understand the needs & expectations of the system users in provinces. *Tanzania *[18] - Visiting each district before the rollout to understand organizational structures, analyze business flow, map key stakeholders at the leadership level, and identify appropriate personnel for training. *Uganda *[31] - Utilization of guidance documents for the programmatic process. *Nigeria *[45] *Capacity strengths:* - Peer mentorship and training by the Technical Working Group as a catalyst. *Kenya *[24] *-* Create a critical mass of local trainers and users of the HRH information system who can help each other. *Uganda *[31]
Maintenance stage	Governance aspect *Structural weakness:* - No policies to mandate data entry and data use, *Indonesia *[49] - The collection and generation of HRH information relied on staff funded by the Global Fund, not by the government, which raises concerns about the sustainability of the system after the funding ceases. *Swaziland *[54]	Governance aspect *Process strengths:* - Development and usage of the sustainability guide and implementation guide. *Nigeria *[45] *Capacity strengths:* - On-site supportive supervision and mentoring to clean up the database to improve the quality. *Uganda *[31] - Organizing training on the usage of data from HRH information systems for better HRH planning, development, and management. *Tanzania *[18]
Environmental factors		
Not stage-specific	Governance aspect *Structural threats:* - Weak leadership & political commitment. *SEARC *[57] - Weak inter-sectoral coordination. *SEARC *[57] - Insufficient financing mechanisms for infrastructure and internet costs due to the decentralization of regulatory functions. *Kenya *[26] - The local government did not allocate funds to maintain the functioning of the HRH information system. *Nigeria *[46] *Capacity threats:* - Poor allocated/devoted budget. *SEARC *[57]*, Bangladesh *[43]*, Ethiopia *[48]*, Pakistan *[51]*, Uganda *[30] - Considerable cost of supportive visits. *Tanzania *[18] - Unavailability of financial support, besides the limited existing development partners, for the HRH information systems. *Kenya *[24] - Geographical uneven distribution of development partners’ financial or technical support within the country. *Kenya *[24]	Governance aspect *Structural opportunities:* - Strong leadership & political commitment. *Kenya *[19]*, Nigeria *[45]*, Sudan *[53] - Dialogue between decision-makers and HRH information systems’ implementers. *Uganda *[30] - Intersectoral collaboration and involvement of key stakeholders. *Kenya *[24]*, Mozambique *[41]*, Nigeria *[45]—Networking with other actors, such as multistakeholder team, stakeholder’s forum for sensitization. *Iran *[36, 38]*, Nigeria *[45]*, Sudan *[53] - Sharing experiences between countries including South–South cooperation. *Zambia *[55] *Capacity opportunities:* - Leveraging existing projects such as disease control programs to build capacity to use HRH information systems. *Kenya *[19] -Financial support from development partners. *DRC *[47]*, Kenya *[19–21]*, Mozambique *[41]*, Nigeria *[45]*, Sudan *[53]*, Tanzania *[18]*, Uganda *[28, 29]*, Zambia *[55]*, Zimbabwe *[56] - Technical support from development partners to manage HRH information systems. *DRC *[47]*, Mozambique *[41]*, Sudan *[53]*, Tanzania *[18]*, Zambia *[55]
Development stage	–	–
Pilot stage	–	–
Rollout stage	Governance aspect *Structural threat:* -Difficulties in collecting data from private and faith-based organization facilities. *Tanzania *[39] *Process threat:* - Government bureaucracy. *Uganda *[30]	Governance aspect *Structural opportunity:* - Decentralization. *Uganda *[30] - Securing support from development partners based on an estimation of the costs required for nationwide rollout. *Uganda* [31]
*Maintenance stage*	Governance aspect *Structural threats:* Decentralization-induced fragmentation of a previously centralized HRIS, limiting its use to central hospitals and excluding district-level facilities. *Malawi *[50] *Process threat:* - Non-use of output from HRH information systems for policymaking. *Iran *[38] *Capacity threat:* - High Cost of server maintenance. *DRC *[47]*, Tanzania y*[18]	Governance aspect *Structural opportunity:* -Decentralized district reorganized the budget to cater to the subscription fees. *Uganda *[31]

-: no specific information mentioned in the reviewed studies

#### Information technology (IT) and infrastructural factors

Thirteen studies and gray literature mentioned the barriers or facilitators related to this category. Office space and the internet connection were recognized as either non-stage-specific or stage-specific barriers when inadequate and as facilitators when adequate. Interoperability among HRH information systems was identified as one of the non-stage-specific facilitators in studies from Mozambique, Tanzania, and Uganda. The Tanzanian study emphasized the importance of interoperability, especially during the development stage.

#### Individual factors

Fifteen studies and gray literature identified the individual factors. Widespread barriers in this category were low levels of IT literacy among users, a lack of motivation to use IT, and high turnover of persons in charge of HRH management. The major facilitators were the system users’ positive attitudes and commitment, including their willingness to learn how to use the system and data.

#### Organizational factors

We define organizational factors as internal factors within organizations related to HRH information systems. Thus, we considered barriers as weaknesses and facilitators as strengths of the relevant organizations. Twenty-seven studies and gray literature mentioned organizational factors, which were further divided into governance and technical aspects per stage. Within the governance aspect, three types of non-stage-specific weaknesses were identified: (1) structural weakness, such as unclear responsibilities of stakeholders and lack of clear strategic plans, protocols, or tools for HRH information systems; (2) process weakness, such as weak communication, cooperation, coordination among concerned parties, and reluctance to share data among stakeholders; and (3) capacity weakness, such as lack of skills to manage the system, because of organizational causes rather than individual causes. The structural weakness identified at the rollout stage was the limited authority given to district stakeholders to manage the system in a local context.

On the other hand, without a specified stage, structural strengths were identified as the establishment of HRH governance structures at the national and subnational levels, their strong leadership to involve relevant stakeholders and policymakers, and well-developed plans for HRH information systems. Established communication mechanisms of governance structures and agreement on interoperability of the systems were identified as process strengths, and supportive supervision as capacity strengths. The existence of local experts or quality control units was a technical strength. In the development and rollout stages, the prior situational analysis was a process strength. Training on the data usage of the HRH information system for better HRH management was identified as a capacity strength at the maintenance stage.

#### Environmental factors

We define environmental factors as external factors for stakeholders in HRH information systems, such as the political, economic, and social context. Thus, we considered barriers as threats and facilitators as opportunities. Twenty-five studies and gray literature reported environmental factors. All these were categorized as governance aspects. Weak leadership, poor inter-sectoral coordination, and financial constraints were the main structural and capacity threats without stage specification. Government bureaucracy was a threat at the rollout stage, and the high cost of server maintenance was a threat at the maintenance stage. While the HRH information system in decentralized settings faced structural threats—such as insufficient financial investment from local governments and misaligned systems between central and local levels that impeded smooth data transmission—one study found that decentralization also served as an opportunity. In that case, districts reorganized their budgets to maintain the HRH information systems. Strong political commitment, networking with other actors, and financial and technical support from development partners were major non-stage-specific opportunities. No study reported social and cultural factors.

### Policy impacts/outcomes of the implementation of digitalized HRH information systems

Sixteen studies and gray literature from nine countries reported the policy impacts or outcomes (Table 4). Five countries reported that the digitalized HRH information system rendered the decision-making process of HRH management more strategic, which was “a new way of doing business” in the case of Uganda. Four countries reported that the HRH gap analysis and forecasting, conducted using the system, contributed to the development of their HRH plans. Staff and budgets were reallocated to optimize available resources in four countries, including the extension of the mandatory retirement age in Kenya. The digitalized HRH information system has been recognized as a powerful tool for policy advocacy in Kenya and Sudan, and for policy evaluation in Uganda. The professional license registration process stimulated transparency in three countries, including Uganda, where one gray literature reported the reduction of malpractice cases. Training institutions were decentralized in Sudan.

**Table 4:** Policy impacts/outcomes of implementation of the digitalized HRH information systems

Impacts/outcomes	Study (country)
*Enhanced overall HRH management*	
HRH managers utilized the analytical results from the HRH information system to inform their strategic decision-making process, including HRH recruitment, selection, allocation and deployment, layoffs or dismissals, capacity building/development, and payroll, as a new approach to “doing business”	Bangladesh [43], DRC [47], Kenya [20], Mozambique [41], Uganda [28, 30]
*HRH and health sector planning*	
HRH information systems facilitated the development of HRH plans, instrumental in addressing staffing gaps, forecasting staffing needs, and informing resource allocation decisions	Kenya [19, 24], Sudan [53], Tanzania [18], Uganda [28, 30]
*Allocation or reallocation of resources*	
By providing accurate data and insights, HRH information systems enabled strategic recruitment and redistribution of staff in underserved areas, optimized the use of available resources, and supported advocacy for additional budget allocation for HRH	DRC [47], Kenya [20, 24], Uganda [29], Nigeria [46]
*Regulatory changes in the mandatory retirement age for nurses and midwives*	
Based on the forecast of workforce shortages by the HRH information system, the civil compulsory service retirement age was officially extended from 55 to 60 years to optimize resource allocation	Kenya [20]
*HRH information systems as a powerful advocacy tool in general*	
The analytical data from HRH information systems contributed to bringing political attention to HRH issues and facilitated resource mobilization	Kenya [24], Sudan [53]
*Professional standards assurance and compliance*	
Where the HRH information systems are run by professional councils to manage licensing and registration, these systems contribute to streamlining, enforcing, or improving health professional licensing and registration processes, and/or enhance the detection of fraudulent applications during recruitment. It also reduced the time spent on regulatory service provision, such as license registration/renewal, and increased revenues from commission fees for professional bodies A mobile telephone reference directory based on the HRH information system enabled the public to access qualification information on each health facility and health professional, thereby reducing malpractice cases, supporting timely license renewals, and facilitating efficient recruitment—ultimately contributing to improved healthcare quality	Kenya [20, 25, 26], Uganda [28], Zambia [55], Uganda [31]
*Decentralization of training institutions*	
The analytical data from HRH information systems informed the political decision-making process to decentralize training institutions for nurses, midwives, and allied health professionals	Sudan [53]
*HRH information systems as an unavoidable tool for policy evaluation*	
HRH information systems are gaining recognition as a valuable tool for evaluating the effectiveness of HRH policies and strategies, particularly in the areas of nursing and midwifery	Uganda [28]

## Discussion

This scoping review critically examined the latest status of digitalized HRH information systems in LMICs in Africa and Asia, focusing on their functional components and linkage with other information systems, barriers and facilitators to their implementation, and their impacts/outcomes on political measures addressing HRH challenges. We reviewed 40 studies and gray literature from 26 LMICs in Africa and Asia, most of which were published from 2007 to 2023. In our review, African countries initiated the implementation of digitalized HRH information systems as early as 2002, and most of their systems were in the rollout stage by the time the study was published. Fewer studies and gray literature were identified in Asian countries, and their implementation stage remains unclear. This regional disparity may partly reflect the relatively stronger engagement of development partners in Africa compared to Asia. Among Asian countries, Indonesia had a well-developed digitalized HRH information system with strong governance and leadership.

Most government-run digitalized HRH information systems exclusively covered health workers in the public sector. Identified barriers and facilitators were often applicable in all implementation stages, and most of them were common across the countries. Some factors appeared to play a more significant role in earlier stages, such as IT and infrastructure; the barriers encountered during these stages had a prolonged negative impact on sustainable use in later stages. Interoperability was identified as a non-stage-specific process strength in the governance aspect for organizational factors, as well as a facilitator in IT factors; lack of interoperability was recognized as a challenge to improved HRH management and evidence-based HRH policymaking. Support from development partners has also been recognized as a facilitator under environmental factors. Some positive impacts of digitalized HRH information systems on policymaking in response to HRH challenges were identified.

This is the first scoping review of digitalized HRH information systems that uses a methodology to stratify the identified barriers and facilitators of implementation into four factors (IT/infrastructure, individual, organizational, and environmental) and four stages (development, pilot, rollout, and maintenance). Many barriers were identified in organizational and environmental factors, particularly in governance. Commonly identified barriers and facilitators in cross-cutting stages were consistent with the previous reviews [9, 10, 58]. Facilitators do not always emerge as a strength in the following stages. For example, training for IT staff was considered a facilitator at any stage, but the relocation of trained and skilled staff became a barrier, especially during the rollout stage. This analytical framework on barriers and facilitators applied for this scoping review (Table 3) can be used in any LMICs to evaluate the implementation and sustainable utilization of HRH information systems.

Although few studies in our scoping review explicitly reported the interoperability between HRH information systems in the country, the lack of interoperability among information systems in general leads to patchy, duplicated, and disorganized information. This not only results in the waste of limited resources, but more critically, hinders the conduct of comprehensive data analysis to develop evidence-based policies [59]. Our review reveals that many countries have multiple HRH information systems with various functional components, each owned by different stakeholders, and these systems have been developed asynchronously for different purposes. In one country where over 30 HRH information systems coexist, the importance of rules and mechanisms to ensure interoperability was finally beginning to be recognized when some systems underwent digitalization [18]. The experiences of such countries can be shared with others as valuable lessons.

The roles and responsibilities of development partners should not be overlooked. Our review identified many LMICs that received long-term technical and financial support from a variety of external sources. This means that the influence of development partners on the digitalization of HRH information systems is significant [42]. Development partners involving any component of the HRH information system must understand the comprehensive picture of the information systems in the country and respect the government’s long-term strategic plans for HRH and digital health. Aligning the direction of external support with the government’s long-term strategic plan would contribute to the achievement of evidence-based policy decisions.

The findings of positive impact on policymaking by the digitalized HRH information system are encouraging. These include contributions to strategic HRH planning and management, reallocation of resources, strengthening the professional license and registration systems, and decentralization of training institutions. These results are particularly notable in contrast to the well-known issue of data underutilization in LMICs [60]. As identified in our scoping review, key factors contributing to this success primarily include governance strength and opportunities, such as political commitment, strong leadership at both national and subnational levels, and effective coordination mechanisms among national stakeholders and development partners.

Finally, more attention should be given to the private sector and migration of HRH information systems in LMICs. With the rapid growth of the private health sector in LMICs, the government’s role in overseeing the private sector, including HRH, is increasingly important [4]. Our scoping review identified that at least two countries have national digitalized HRH information systems covering both the public and private sectors. In other countries, HRH information in the private sector is often covered by non-governmental sector systems; however, interoperability with government-owned systems is not always guaranteed. With regard to HRH migration, it poses one of the major threats to achieving universal health coverage in LMICs. Since 2010, all countries have been encouraged to gather migration-related data [61], yet our scoping review identified only three countries with the component of migration in their HRH information systems. The possibility of HRH migration has increased because of political and economic environmental changes. These include prolonged conflicts and instability in some geographic regions, global economic downturns, and widening disparities in payment and working conditions between countries. The COVID-19 pandemic was a good example showing how health threats intensify the recruitment of health workers from LMICs to high-income countries, after health workers in high-income countries left for other sectors due to safety concerns [5, 62].

## Limitations

This scoping review was limited to studies published in the English language. A broader examination of the literature published in other languages may yield more accurate results. Publication bias is a risk because most published studies report only positive results and are compiled by the implementing organization or development partner. The stages in this review correspond to those mentioned at the time of publication. Consequently, certain countries may have progressed to subsequent stages, such as from development to pilot or rollout stages.

## Conclusion

The scoping review identified the implementation status of the digitalized HRH information systems in LMICs, their components and linkages with other health information systems, facilitators and barriers to their implementation, and policy impacts or outcomes. Our methodology for analyzing facilitators and barriers, which stratifies them into four factors and four stages, can be used as an analytical framework to evaluate HRH information systems in any country. Many barriers were identified in organizational and environmental factors, particularly regarding governance. Interoperability among multiple HRH information systems within a country is the key facilitator, in which development partners play a critical role. Political commitment, strong leadership at both national and subnational levels, and effective coordination mechanisms among national stakeholders and development partners were key to achieving policy impact. Data on the private sector and migration could be further strengthened as system components.

## Supplementary Information

Additional file 1.

Additional file 2.

## Data Availability

No datasets were generated or analyzed during the current study.

## Abbreviations

CDC: United States Centers for Disease Control and Prevention
DRC: Democratic Republic of Congo
HRH: Human Resources for Health
IT: Information technology
JICA: Japan International Cooperation Agency
LMICs: Low- and middle-income countries
PEPFAR: US President’s Emergency Plan for AIDS Relief
PRISMA-ScR: Preferred Reporting Items for Systematic reviews and Meta-Analyses extension for Scoping Reviews
SEAR: WHO South-East Asia region countries
UHC: Universal health coverage
USAID: United States Agency for International Development
WHO: World Health Organization
