# Supplementary material for: Digitalized human resources for health information systems in low- and middle-income countries: a scoping review
Source: Hum Resour Health. 2025 Dec 19;24:3. doi: 10.1186/s12960-025-01043-x (PMC12809960; doi:10.1186/s12960-025-01043-x)
Supplement: Supplementary file 2 — Additional file 2. [file 12960_2025_1043_MOESM2_ESM.docx]

**Search terms for databases**

| 1 | "Human resource information system*"[Title/Abstract] OR "Human resources information system*"[Title/Abstract] OR "HR information system*"[Title/Abstract] OR "HRIS"[Title/Abstract] OR "iHRIS"[Title/Abstract] |
| --- | --- |
| 2 | (((Health workforce information system[Title/Abstract]) OR (Health workforce information systems[Title/Abstract])) OR (Health work force information system[Title/Abstract])) OR (Health work force information systems[Title/Abstract]) |
| 3 | (((health workforce registry[Title/Abstract]) OR (health workforce registries[Title/Abstract])) OR (health work force registry[Title/Abstract])) OR (health work force registries[Title/Abstract]) |
| 4 | ("human resource for health"[Title/Abstract] OR "human resources for health"[Title/Abstract] OR "HRH"[Title/Abstract]) AND "observatory"[Title/Abstract] |
| 5 | "Human resource management system*"[Title/Abstract] OR "Human resources management system*"[Title/Abstract] OR "Electronic human resource management"[Title/Abstract] OR "e-HRM"[Title/Abstract] OR "eHRM"[Title/Abstract] OR "workforce surveillance"[Title/Abstract] OR "workforce database"[Title/Abstract] |
| 6 | 1 OR 2 OR 3 OR 4 OR 5 |
| 7 | "Health Workforce"[MeSH Terms] OR "Health personnel"[MeSH Terms] OR "Health Workforce"[Title/Abstract] OR "Health personnel"[Title/Abstract] OR "HRH"[Title/Abstract] OR "Human resources for health"[Title/Abstract] OR "Human resource for health"[Title/Abstract] |
| 8 | "Health information systems"[MeSH Terms] OR "Health management information system"[Title/Abstract] OR "information system*"[Title/Abstract] OR "management system*"[Title/Abstract] OR "Information technology"[Title/Abstract] OR "Management technology"[Title/Abstract] OR "regist*"[Title/Abstract] OR "informatics"[Title/Abstract] OR "database"[Title/Abstract] |
| 9 | 7 AND 8 |
| 10 | "hospital*"[Title/Abstract] OR "clinic*"[Title/Abstract] OR "health facilit*"[Title/Abstract] OR "health center*"[Title/Abstract] OR "patient*"[Title/Abstract] OR "client*"[Title/Abstract] OR "medical record"[Title/Abstract] OR "admission"[Title/Abstract] OR "ward"[Title/Abstract] OR "tally"[Title/Abstract] OR "community"[Title/Abstract] OR "outreach"[Title/Abstract] OR "volunteer"[Title/Abstract] OR "infrastructure"[Title/Abstract] OR "equipment*"[Title/Abstract] OR "commodit*"[Title/Abstract] OR "drug"[Title/Abstract] OR "medic*"[Title/Abstract] OR "surveillance"[Title/Abstract] OR "service*"[Title/Abstract] OR "care"[Title/Abstract] OR "treatment"[Title/Abstract] OR "immunization"[Title/Abstract] OR "immunisation"[Title/Abstract] OR "antenatal"[Title/Abstract] OR "delivery"[Title/Abstract] OR "postnatal"[Title/Abstract] OR "malaria"[Title/Abstract] OR "diarrhoea"[Title/Abstract] OR "diarrhea"[Title/Abstract] OR "respiratory"[Title/Abstract] OR "infections"[Title/Abstract] OR "HIV"[Title/Abstract] OR "tuberculosis"[Title/Abstract] OR "blood pressure"[Title/Abstract] OR "hypertension"[Title/Abstract] OR "diabetes"[Title/Abstract] OR "surgery"[Title/Abstract] OR "trauma"[Title/Abstract] |
| 11 | 9 NOT 10 |
| 12 | 6 OR 11 |
| 13 | "developing countries"[MeSH Terms] OR ("developing countr*"[Title/Abstract] OR "middle income countr*"[Title/Abstract] OR "low income countr*"[Title/Abstract] OR "LMIC"[Title/Abstract] OR "LMICs"[Title/Abstract]) OR ("afghan*"[Title/Abstract] OR "benin*"[Title/Abstract] OR "burkin*"[Title/Abstract] OR "burundi*"[Title/Abstract] OR "central african*"[Title/Abstract] OR "Chad"[Title/Abstract] OR "chadian*"[Title/Abstract] OR "Comoros"[Title/Abstract] OR "comorian*"[Title/Abstract] OR "comoran*"[Title/Abstract] OR "congo*"[Title/Abstract] OR "eritrea*"[Title/Abstract] OR "ethiopia*"[Title/Abstract] OR "gambia*"[Title/Abstract] OR "guinea*"[Title/Abstract] OR "haiti*"[Title/Abstract] OR "north korea*"[Title/Abstract] OR "democratic people s republic of korea"[Title/Abstract] OR "liberia*"[Title/Abstract] OR "Madagascar"[Title/Abstract] OR "malagasy*"[Title/Abstract] OR "malawi*"[Title/Abstract] OR "mali*"[Title/Abstract] OR "mozambiqu*"[Title/Abstract] OR "mozambican*"[Title/Abstract] OR "nepal*"[Title/Abstract] OR "niger*"[Title/Abstract] OR "rwanda*"[Title/Abstract] OR "senegal*"[Title/Abstract] OR "sierra leon*"[Title/Abstract] OR "somali*"[Title/Abstract] OR "sudan*"[Title/Abstract] OR "tanzania*"[Title/Abstract] OR "togo*"[Title/Abstract] OR "uganda*"[Title/Abstract] OR "zimbabw*"[Title/Abstract]) OR ("angola*"[Title/Abstract] OR "armenia*"[Title/Abstract] OR "bangladesh*"[Title/Abstract] OR "bhutan*"[Title/Abstract] OR "bolivia*"[Title/Abstract] OR "cabo verd*"[Title/Abstract] OR "cambodia*"[Title/Abstract] OR "cameroon*"[Title/Abstract] OR "congo*"[Title/Abstract] OR "cote d ivoir*"[Title/Abstract] OR "ivory coast"[Title/Abstract] OR "ivorian*"[Title/Abstract] OR "djibout*"[Title/Abstract] OR "egypt*"[Title/Abstract] OR "salvador*"[Title/Abstract] OR "georgia*"[Title/Abstract] OR "ghana*"[Title/Abstract] OR "guatemala*"[Title/Abstract] OR "hondura*"[Title/Abstract] OR "india*"[Title/Abstract] OR "indonesia*"[Title/Abstract] OR "jordan*"[Title/Abstract] OR "kenya*"[Title/Abstract] OR "Kiribati"[Title/Abstract] OR "kosov*"[Title/Abstract] OR "kyrgyz*"[Title/Abstract] OR "Lao"[Title/Abstract] OR "Laos"[Title/Abstract] OR "laotian*"[Title/Abstract] OR "lesoth*"[Title/Abstract] OR "basotho*"[Title/Abstract] OR "mauritania*"[Title/Abstract] OR "micronesia*"[Title/Abstract] OR "moldov*"[Title/Abstract] OR "mongolia*"[Title/Abstract] OR "Mongols"[Title/Abstract] OR "Mongol"[Title/Abstract] OR "morocc*"[Title/Abstract] OR "myanmar*"[Title/Abstract] OR "Burmese"[Title/Abstract] OR "burman*"[Title/Abstract] OR "nicaragua*"[Title/Abstract] OR "nigeria*"[Title/Abstract] OR "pakistan*"[Title/Abstract] OR "papua*"[Title/Abstract] OR "philippin*"[Title/Abstract] OR "filipin*"[Title/Abstract] OR "sao tome*"[Title/Abstract] OR "solomon island*"[Title/Abstract] OR "sri lank*"[Title/Abstract] OR "swazi*"[Title/Abstract] OR "syria*"[Title/Abstract] OR "tajik*"[Title/Abstract] OR "timor*"[Title/Abstract] OR "tunisia*"[Title/Abstract] OR "ukrain*"[Title/Abstract] OR "uzbek*"[Title/Abstract] OR "vanuatu*"[Title/Abstract] OR "vietnam*"[Title/Abstract] OR "west bank"[Title/Abstract] OR "Gaza"[Title/Abstract] OR "palestin*"[Title/Abstract] OR "yemen*"[Title/Abstract] OR "zambia*"[Title/Abstract]) OR ((("albania*"[Title/Abstract] OR "algeria*"[Title/Abstract] OR "samoa*"[Title/Abstract] OR "argentin*"[Title/Abstract] OR "azerbaijan*"[Title/Abstract] OR "belarus*"[Title/Abstract] OR "belize"[Title/Abstract] OR "bosnia*"[Title/Abstract] OR "herzegovin*"[Title/Abstract] OR "botswana*"[Title/Abstract] OR "batswana*"[Title/Abstract] OR "brazil*"[Title/Abstract] OR "bulgaria*"[Title/Abstract] OR "Chinese"[Title/Abstract] OR "China"[Title/Abstract] OR "colombia*"[Title/Abstract] OR "costa rica*"[Title/Abstract] OR "cuba*"[Title/Abstract] OR "dominica*"[Title/Abstract] OR "guinea*"[Title/Abstract] OR "ecuador*"[Title/Abstract] OR "Eswatini"[Title/Abstract] OR "Swaziland"[Title/Abstract]) AND ("Or"[All Fields] AND "Swazi"[Title/Abstract])) OR "Swazis"[Title/Abstract] OR "fiji*"[Title/Abstract] OR "gabon*"[Title/Abstract] OR "Grenada"[Title/Abstract] OR "grenadian*"[Title/Abstract] OR "guyana*"[Title/Abstract] OR "Guyanese"[Title/Abstract] OR "iran*"[Title/Abstract] OR "persia*"[Title/Abstract] OR "iraq*"[Title/Abstract] OR "jamaica*"[Title/Abstract] OR "kazakh*"[Title/Abstract] OR "Lebanon"[Title/Abstract] OR "Lebanese"[Title/Abstract] OR "libya*"[Title/Abstract] OR "macedonia*"[Title/Abstract] OR "malaysia*"[Title/Abstract] OR "maldiv*"[Title/Abstract] OR "marshall island*"[Title/Abstract] OR "Marshallese"[Title/Abstract] OR "maurit*"[Title/Abstract] OR "mexic*"[Title/Abstract] OR "montenegr*"[Title/Abstract] OR "namibia*"[Title/Abstract] OR "nauru*"[Title/Abstract] OR "paraguay*"[Title/Abstract] OR "peru*"[Title/Abstract] OR "russia*"[Title/Abstract] OR "serbia*"[Title/Abstract] OR "Serbs"[Title/Abstract] OR "south africa*"[Title/Abstract] OR "lucia*"[Title/Abstract] OR "saint vincent"[Title/Abstract] OR "grenadin*"[Title/Abstract] OR "Vincentian"[Title/Abstract] OR "suriname*"[Title/Abstract] OR "Thailand"[Title/Abstract] OR "Thai"[Title/Abstract] OR "tonga*"[Title/Abstract] OR "Turkey"[Title/Abstract] OR "Turkish"[Title/Abstract] OR "turkmen*"[Title/Abstract] OR "tuvalu*"[Title/Abstract] OR "venezuela*"[Title/Abstract]) OR ("afghanistan"[MeSH Terms:noexp] OR "albania"[MeSH Terms:noexp] OR "algeria"[MeSH Terms:noexp] OR "american samoa"[MeSH Terms:noexp] OR "angola"[MeSH Terms:noexp] OR "argentina"[MeSH Terms:noexp] OR "armenia"[MeSH Terms:noexp] OR "azerbaijan"[MeSH Terms:noexp] OR "bangladesh"[MeSH Terms:noexp] OR "benin"[MeSH Terms:noexp] OR "republic of belarus"[MeSH Terms:noexp] OR "belize"[MeSH Terms:noexp] OR "bhutan"[MeSH Terms:noexp] OR "bolivia"[MeSH Terms:noexp] OR "bosnia and herzegovina"[MeSH Terms:noexp] OR "botswana"[MeSH Terms:noexp] OR "brazil"[MeSH Terms:noexp] OR "bulgaria"[MeSH Terms:noexp] OR "burkina faso"[MeSH Terms:noexp] OR "burundi"[MeSH Terms:noexp] OR "cambodia"[MeSH Terms:noexp] OR "cameroon"[MeSH Terms:noexp] OR "cabo verde"[MeSH Terms:noexp] OR "central african republic"[MeSH Terms:noexp] OR "Chad"[MeSH Terms:noexp] OR "China"[MeSH Terms:noexp] OR "colombia"[MeSH Terms:noexp] OR "Comoros"[MeSH Terms:noexp] OR "congo"[MeSH Terms:noexp] OR "costa rica"[MeSH Terms:noexp] OR "cote d ivoire"[MeSH Terms:noexp] OR "cuba"[MeSH Terms:noexp] OR "djibouti"[MeSH Terms:noexp] OR "Democratic Republic of the Congo"[MeSH Terms:noexp] OR "dominica"[MeSH Terms:noexp] OR "dominican republic"[MeSH Terms:noexp] OR "timor leste"[MeSH Terms:noexp]) OR ("ecuador"[MeSH Terms:noexp] OR "egypt"[MeSH Terms:noexp] OR "el salvador"[MeSH Terms:noexp] OR "eritrea"[MeSH Terms:noexp] OR "Eswatini"[MeSH Terms:noexp] OR "equatorial guinea"[MeSH Terms:noexp] OR "ethiopia"[MeSH Terms:noexp] OR "fiji"[MeSH Terms:noexp] OR "gabon"[MeSH Terms:noexp] OR "gambia"[MeSH Terms:noexp] OR "georgia republic"[MeSH Terms:noexp] OR "ghana"[MeSH Terms:noexp] OR "Grenada"[MeSH Terms:noexp] OR "guatemala"[MeSH Terms:noexp] OR "guinea"[MeSH Terms:noexp] OR "guinea bissau"[MeSH Terms:noexp] OR "guyana"[MeSH Terms:noexp] OR "haiti"[MeSH Terms:noexp] OR "honduras"[MeSH Terms:noexp] OR "india"[MeSH Terms:noexp] OR "indonesia"[MeSH Terms:noexp] OR "iran"[MeSH Terms:noexp] OR "iraq"[MeSH Terms:noexp] OR "jamaica"[MeSH Terms:noexp] OR "jordan"[MeSH Terms:noexp] OR "kazakhstan"[MeSH Terms:noexp] OR "kenya"[MeSH Terms:noexp] OR "democratic people s republic of korea"[MeSH Terms:noexp] OR "kosovo"[MeSH Terms:noexp] OR "kyrgyzstan"[MeSH Terms:noexp] OR "Laos"[MeSH Terms:noexp] OR "Lebanon"[MeSH Terms:noexp] OR "lesotho"[MeSH Terms:noexp] OR "liberia"[MeSH Terms:noexp] OR "libya"[MeSH Terms:noexp] OR "republic of north macedonia"[MeSH Terms:noexp] OR "Madagascar"[MeSH Terms:noexp] OR "malaysia"[MeSH Terms:noexp] OR "malawi"[MeSH Terms:noexp] OR "mali"[MeSH Terms:noexp] OR "mauritania"[MeSH Terms:noexp] OR "mauritius"[MeSH Terms:noexp] OR "melanesia"[MeSH Terms:noexp] OR "mexico"[MeSH Terms:noexp] OR "micronesia"[MeSH Terms:noexp] OR "moldova"[MeSH Terms:noexp]) OR ("mongolia"[MeSH Terms:noexp] OR "montenegro"[MeSH Terms:noexp] OR "morocco"[MeSH Terms:noexp] OR "mozambique"[MeSH Terms:noexp] OR "myanmar"[MeSH Terms:noexp] OR "namibia"[MeSH Terms:noexp] OR "nepal"[MeSH Terms:noexp] OR "nicaragua"[MeSH Terms:noexp] OR "niger"[MeSH Terms:noexp] OR "nigeria"[MeSH Terms:noexp] OR "pakistan"[MeSH Terms:noexp] OR "papua new guinea"[MeSH Terms:noexp] OR "paraguay"[MeSH Terms:noexp] OR "peru"[MeSH Terms:noexp] OR "philippines"[MeSH Terms:noexp] OR "russia"[MeSH Terms:noexp] OR "russia pre 1917"[MeSH Terms:noexp] OR "rwanda"[MeSH Terms:noexp] OR "saint lucia"[MeSH Terms:noexp] OR "Saint Vincent and the Grenadines"[MeSH Terms:noexp] OR "samoa"[MeSH Terms:noexp] OR "sao tome and principe"[MeSH Terms:noexp] OR "senegal"[MeSH Terms:noexp] OR "serbia"[MeSH Terms:noexp] OR "montenegro"[MeSH Terms:noexp] OR "sierra leone"[MeSH Terms:noexp] OR "sri lanka"[MeSH Terms:noexp] OR "somalia"[MeSH Terms:noexp] OR "south africa"[MeSH Terms:noexp] OR "south sudan"[MeSH Terms:noexp] OR "sudan"[MeSH Terms:noexp] OR "suriname"[MeSH Terms:noexp] OR "Eswatini"[MeSH Terms:noexp] OR "syria"[MeSH Terms:noexp] OR "tajikistan"[MeSH Terms:noexp] OR "tanzania"[MeSH Terms:noexp] OR "Thailand"[MeSH Terms:noexp] OR "togo"[MeSH Terms:noexp] OR "tonga"[MeSH Terms:noexp] OR "tunisia"[MeSH Terms:noexp] OR "Turkey"[MeSH Terms:noexp] OR "turkmenistan"[MeSH Terms:noexp] OR "uganda"[MeSH Terms:noexp] OR "ukraine"[MeSH Terms:noexp] OR "uzbekistan"[MeSH Terms:noexp] OR "vanuatu"[MeSH Terms:noexp] OR "venezuela"[MeSH Terms:noexp] OR "vietnam"[MeSH Terms:noexp] OR "yemen"[MeSH Terms:noexp] OR "zambia"[MeSH Terms:noexp] OR "zimbabwe"[MeSH Terms:noexp]) |
| 14 | 12 AND 13 |
